# Two-Stage Surgical Procedure in Intra-articular Distal Tibiofibular Fractures with Soft Tissue Injury: in Which Stage Should the Fibular Plate be Applied at Initial Surgery?

**DOI:** 10.5704/MOJ.2011.014

**Published:** 2020-11

**Authors:** A Yuce, SS Dedeoglu, Y Imren, M Yerli, H Gurbuz

**Affiliations:** 1 Department of Orthopaedic and Traumatology, Basaksehir Pine and Sakura City Hospital, Istanbul, Turkey; 2 Department of Orthopaedic and Traumatology, Prof. Dr. Cemil Tascıoglu City Hospital, Istanbul, Turkey

**Keywords:** intra-articular fracture, distal tibia, two-stage surgery, fibular plating, external fixation

## Abstract

**Introduction:**

The selection of the stage where fibular plate was performed in two-stage surgery of the intra-articular distal tibiofibular fractures with soft tissue injury is still controversial. The aim of the study was to compare the complications, radiological and functional outcomes between the patients who had fibular plate at initial or second phase during surgical management of such fractures.

**Materials and Methods:**

In this study, medical records of 47 patients who underwent a two-stage surgical procedure for intra-articular distal tibia fractures accompanying soft tissue injury were retrospectively examined. Delta frame was applied in all cases within 24 hours following admission to the emergency department in accordance with AO principles. Those cases where fibular plate was applied during the initial stage and the second stage were classified as Group 1 and Group 2 in order to compare recorded data between the two groups.

**Results:**

According to the results of the study, there were 25 cases in Group 1 and 22 cases in Group 2 in which fibular plate was applied at the first stage and the second stage, respectively. The mean follow-up was found as 27.7±7.0 months in Group 1 and 28.2±6.2 months in Group 2 (p=0.778). No difference was found between the two groups in terms of the age, sex, hospital stay, the time between two surgical procedures, tibiofibular angle and AOFAS scoring (p>0.05).These two groups were also similar in mechanism of injury, Denise-Weber or AO classification, rates of tibiofibular malalignment on post-operative CT, fibular rotation, intra-articular tibial step-off, tibial varus-valgus duration of union, rate of infection, fibular angulation and the presence of the flap/graft/debridement (p>0.05).

**Conclusion:**

In conclusion, two-stage surgical procedure in intra-articular distal tibiofibular fractures may be an effective method decreasing soft tissue complications. The timing of the open reduction and internal fixation of the fibula at different stages may not necessarily have an impact on the success of the post-operative tibial reduction, the total duration of surgery, syndesmosis malalignment or soft tissue complications.

## Introduction

Distal tibia fractures constituted 7 to 10% of all tibia fractures and up to 80% of them were reported to be associated with ipsilateral fibula fractures^1,2^. Having experienced after high-energy injuries, these fractures were commonly complicated with soft tissue injury, which may result from the destructive impact of the fracture transmitted to soft tissues. Closed tibia fractures may often accompany intensive contusions, fracture bullae, or severe muscle injury^3^.

The aim of the therapy in intra-articular distal tibiofibular (IDTF) fractures include restoring the joint and ankle functions, ensuring appropriate fracture union, anatomically reducing all fracture fragments, and managing soft tissues^4,5^. The most appropriate treatment modality for IDTF fracture is controversial since there is not a single universally accepted method^6,7^. The most important factors associated with diminished wound complications are the recognition of soft tissue injury, appropriate surgical timing, and the application of a surgical procedure considering soft tissue healing. Therefore, surgeons mostly prefer two-stage surgical protocol designed to support healing of traumatised soft tissue before the definitive fixation8.

In two-stage surgery, osteosynthesis of fibula fracture with plate is recommended for the preservation of tibial length and prevention of valgus malunion and rotation forces^2,9,10^. It is controversial to determine the stage when the fibula should be fixed^11,12^. Reduction of fibula with external fixation may lead to decrease in the lateral column of the tibia at the first stage. Moreover, it indirectly provides reduction of Volkmann and Chaput fragments via ligamentotaxis^12^. This may facilitate joint reduction and axial plan reduction in a permanent treatment^13^. On the other hand, unplanned skin incision performed for fibula fixation at the first stage could pose a risk for the skin in the following step^11,12^. In addition, in case of comminuted fibula fracture or a bone defect, non-anatomical fibula fixation performed at the first stage may prevent anatomical tibia reduction during the permanent surgery^12^.

It is believed that fibula should be fixed with an external fixator in the first stage in distal tibiofibular fractures accompanying soft tissue injury. It is considered that the performance of this fixation together with the external fixator in the first stage does not increase soft tissue complications and facilitates joint reduction of the tibia. Our hypothesis is that the application of fibular fixation in the first stage in two-stage surgery provides better tibial fracture reduction with ligamentotaxis and is associated with short surgery times. It does not increase either infection or soft tissue complications.

The aim of the present study was to compare post-operative radiologic and functional outcomes and complication rates between patients who underwent fibula fixation at the initial or the second stage in IDTF fractures managed with a two-stage surgery.

## Materials and Methods

A retrospective review of patients who underwent two-stage surgical procedure for soft tissue injury accompanying intra-articular distal tibiofibular fracture between 2013-2018 was conducted. In the first surgery, external fixator was applied and then external fixator was removed. The internal fixation was performed at the same session in the second surgery in 47 cases.

Inclusion criteria of the study were as follows; cases with a follow-up period of more than six months and cases over 18 years of age with IDTF fracture accompanied by distal fibula fracture accompanying grade 2/3 soft tissue injury according to the Tscherne classification.

Exclusion criteria of the study were as follows; cases whose first surgery was performed after 24 hours to the trauma, neurovascular injury with fracture, open fractures, pathological fractures and cases with peripheral vascular disease or diabetic foot neuropathy, cases without internal fixation of fibula fracture and non-plate internal fixation methods in the treatment of fibular fractures.

The patients included in the study were operated by senior surgeons. External fixator (EF) was applied in all cases within the first 24 hours after admission. Delta frame was applied in all cases in accordance with AO principles after the application of one transcalcaneal and two Shanz screws to the tibial shaft by tubular fixator of Tasarımmed company.

Osteosynthesis was performed with plate-screw after open reduction in distal fibula fractures. In osteosynthesis, posterolateral anatomic distal fibula plate of TST company was performed. In cases where the plate was applied at the first stage, the presence of the posterior fragment was considered while deciding on the incision preference. If the posterior fragment fracture contained more than 30% of the joint surface, or if the displacement of this fragment was more than 2mm, a fibula plate was applied through a posterolateral incision in the second surgical intervention because this fragment may need reduction. In all other cases, a fibula fracture was treated using a lateral incision.

According to the fracture pattern, one of the anteromedial or medial approaches was used in the surgery of distal tibial fractures. The fracture was stabilised with medial or anterolateral distal tibia plate according to fracture type and fracture fragments. The fracture fragments were attached with plate to each other with cannula screws prior to plate application when necessary. In the presence of the posterior joint fragment requiring fixation, the fracture fragment in the posterior was stabilised by using posterior plate application or cannulated screws moved from the anterior to posterior.

After the first surgery, the patients were discharged after providing recommendations and they were informed to be called for the controls. Two weeks after the first surgical treatment, the patients were evaluated by the orthopaedic committee and it was decided to proceed with the second stage. In the soft tissue examinations of the patients performed by the orthopaedic committee, it was expected that the bullous skin lesions would heal, the skin folds would become visible again with the ankle movements, the skin tissue under the appropriate necrotic tissues would deform and the edema would not be observed.

The operations were performed by two different surgeons (surgeon 1 and surgeon 2). The same surgeon, who planned and performed the first-stage surgery of the cases, also performed the operations in the second stage. Surgeon 1 operated all patients who had the fibula plate applied in the first stage. Four of the cases where the fibula plate was applied in the second stage were operated by surgeon 1 and the others by surgeon 2. Two-stage surgery was decided by the surgeon performing the operation of the patients. The stage to apply the fibular plate was the choice of the surgeon. The soft tissue criteria used in the 2-stage surgical decision were also used when applying the distal fibula plate. In the presence of soft tissue damage covering the incision area and its surrounding in the lateral ankle position, fibula plate application was left to surgery in Stage 2 regardless of the surgeon's preference.

All patients were applied AFO for two weeks after the definitive surgery. AFO was then removed and non-weight-bearing and ankle movements were initiated. Angulations exceeding 5° in the coronal plane and exceeding 10° in the sagittal plane were regarded as malalignment^5^.

Tibiofibular malalignment was measured as described by Elgafy. Anterior and posterior tibiofibular distances were measured by the lines perpendicularly drawn from distances between fibula and incisura anterior and posterior facets to the joint. In these measurements, tibiofibular malalignment was accepted in cases where the difference between anterior tibiofibular distance and posterior tibiofibular distance was greater than 2mm^14^.

Data on demographic characteristics of the patients, mechanism of injury, duration of follow-up, time between two surgeries, total operation time, union time, AOFAS scoring, tibia fracture type according to AO and Ruedi-Allgower classification, fibula fracture type according to Denis-Weber classification, tibiofibular joint malalignment, fibular shortening and rotation, tibial intra-articular step-off as measured by post-operative computerised tomography (CT), tibial angulation and shortening as measured from anteroposterior and lateral radiographs, and rates of skin flap or grafts, infection, non-union, malunion, and other complications were collected.

**Fig. 1:**
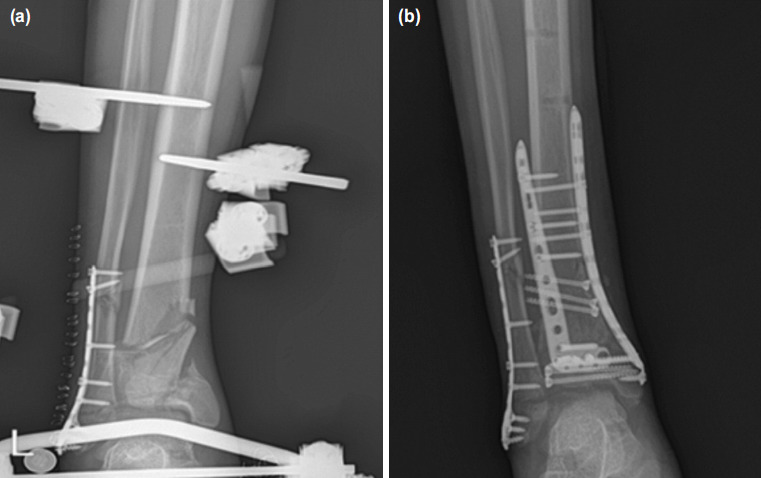
(a) Radiograph images of the patient who underwent fibula fixation in the first stage. Fracture of the fibula was treated with open reduction internal fixation in the first stage. (b) Definitive treatment was applied to the fracture of the tibia after soft tissue swelling resolves.

**Fig. 2:**
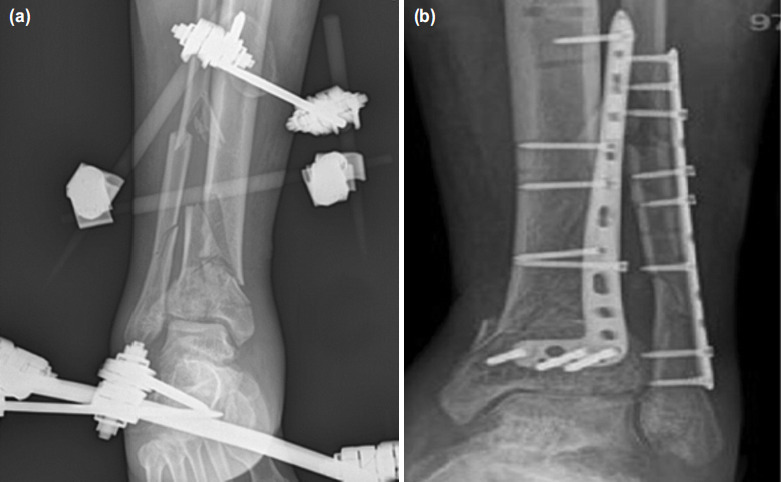
(a) Radiograph images of the patient who did not have fibula fixation in the first stage. In the first stage, only external fixator was applied for fractures of the tibia and fibula. (b) Definitive treatment was applied to the fracture of the tibia and fibula after soft tissue swelling resolves.

The cases where the fibula plate was applied with the external fixator in the first session were named as Group 1 (Fig. 1). The cases who underwent plate to the fracture of the fibula during internal fixation for the treatment of tibial fracture were named as Group 2 (Fig. 2). A statistical comparison of the data recorded between the two groups was carried out.

Statistical analyses were performed through SPSS Windows version 24.0 software package. The continuous and categorical variables in the study were expressed as mean ± standard deviation and numbers and percentages, respectively. The normality of the data was tested with Shaphiro-Wilk test, where comparisons of two independent groups were performed by Student’s t-test or Mann-Whitney U test for normally and non-normally distributed data, respectively. Categorical variables were analysed by Pearson or Fisher’s exact chi-square tests. An overall 5% Type I error level was used to identify the statistical significance.

## Results

Twenty-five cases (16 males and 9 females) in Group 1 and 22 cases (15 males and 7 females) in Group 2 were included in the study. In Group 1, the mean age was 34.4 ± 10.78 years, follow-up time was 27.68 ± 6.96 months, the time between two surgeries was 15.8 ± 3.63 days, total hospitalisation time was 13.44 ± 2.68 days and AOFAS score was found as 72.24 ± 22.19. In Group 2, the mean age was 33.09 ± 10.81, follow-up time was 28.23 ± 6.17 months, the interval between two surgeries was 15.18 ± 4.5 days, total hospitalisation time was 12.05 ± 2.89 days and AOFAS score was found as 71.23 ± 21.25. There was no statistically significant difference between the two groups in terms of mean age, follow-up, time between two surgeries, total hospitalisation time, and AOFAS scores (p> 0.05). Although the mean operation time of the Group 2 (142.50 ± 38.32) was higher than the mean operation time of Group 1 (132.20 ± 46.50), no statistically significant difference was observed (p = 0.416) (Table I).

**Table I:** Comparison of continuous parameters in study groups

Parameters	Group 1 (n+22) x̅ ± SD	Group 2 (n+22) x̅ ± SD	t/z	P
Age (years)	34.4 ± 10.78	33.09 ± 10.81	t=0.415	0.680
Duration of follow-up (months)	27.68 ± 6.96	28.23 ± 6.17	t=-0.283	0.778
Total length of hospitalization (days)	13.44 ± 2.68	12.05 ± 2.89	t=1.718	0.093
Duration between surgeries (days)	15.8 ± 3.63	15.18 ± 4.5	t=0.521	0.605
AOFAS	72.24 ± 22.19	71.23 ± 21.25	z=-0.363	0.717

* t value was calculated by student t-test, and z-value by Mann-Whitney U test. SD, standard deviation.

According to the Tscherne classification, 9 patients (36%) were type 2, 16 patients (64%) were type 3 in Group 1, while 9 patients (40.9%) were type 2, 13 patients (59.1%) were type 3 in Group 2 (p = 0.730). A total of, 80% of the patients had fracture mechanism with high energy trauma in Group 1, while 72.7% of the patients had high energy trauma in Group 2. According to Denis-Weber classification of fibula fracture, 8 cases were B (32%), 17 cases were C (68%) in Group 1. Seven cases were B (31.8%) and 15 cases were C (68.2%) (p=0.814) in Group 2. According to AO fracture classification, 2 cases were B1 (8%), 6 cases were C1 (24%), 8 cases were C2 (32%), 9 cases were C3 (36%) in Group 1. Five cases were C1 (22.7%), 7 cases were C2 (31.8%) and 10 cases were C3 (45.5%) (p=0.666) (Table II).

**Table II:** Comparison of categorical variables in study groups

		Group 1 n (%)	Group 2 n (%)	X^2^	P
Mechanism of injury	Simple fall	5 (20.0)	6 (27.3)	1.406	0.704
	Occupational accident	6 (24.0)	7 (31.8)		
	Motor accident	6 (24.0)	5 (22.7)		
	Fall from height	8 (32.0)	4 (18.2)		
Denis-weber fibula classifications	B	8 (32.0)	7 (31.8)	0.946	0.814
	C	17 (68.0)	15 (68.2)		
Tibia AO classification	B1	2 (8.0)	0 (0.0)	3.222	0.666
	C1	6 (24.0)	5 (22.7)		
	C2	8 (32.0)	7 (31.8)		
	C3	9 (36.0)	10 (45.5)		
Tibia Ruedialgover classification	Type 1	1 (4.0)	0 (0.0)	4.112	0.391
	Type 2	9 (36.0)	9 (40.9)		
	Type 3	15 (60.0)	13 (59.1)		
Fibular rotation	Present	3 (12.0)	3 (13.6)	0.028	0.867
	None	22 (88.0)	19 (86.4)		
Fibular shortening	Present	1 (4.0)	3 (13.6)	1.396	0.237
	None	24 (96.0)	19 (86.4)		
Tibial intra-articular stepping	Present	1 (4.0)	1 (4.5)	0.009	0.926
	None	24 (96.0)	21 (95.5)		
Tibial coronal angulation	2° VALGUS	1 (4.0)	0 (0.0)	7.238	0.405
	2° VARUS	1 (4.0)	0 (0.0)		
	3° VALGUS	1 (4.0)	0 (0.0)		
	3° VARUS	0 (0.0)	1 (4.5)		
	4° VALGUS	0 (0.0)	1 (4.5)		
	5° VARUS	0 (0.0)	1 (4.5)		
	60 VALGUS	0 (0.0)	1 (4.5)		
	None	22 (88.0)	18 (81.8)		
Tibia saggital angulation (degree)	0°	23 (92.0)	19 (86.4)	3.203	0.525
	2°	1 (4.0)	0 (0.0)		
	3°	1 (4.0)	1 (4.5)		
	5°	0 (0.0)	1 (4.5)		
	6°	0 (0.0)	1 (4.5)		
Fibular angulation (degree)	0°	25 (100.0)	20 (90.9)	2.374	0.305
	3°	0 (0.0)	1 (4.5)		
	4°	0 (0.0)	1 (4.5)		

* x2 value was calculated by chi-square test.

Post-operative outcomes were as follows for the groups. Three patients had fibular rotation, one patient had fibular shortening, and one patient had articular step-off in Group 1. Three patients had fibular rotation, three patients had fibular shortening, and one patient had articular step-off in Group 2. Two group were found to be similar in terms of fibular rotation, the presence of fibular shortening and intra-articular step-off (p>0.05) (Table III).

Group 1 had no post-operative coronal deformity of 5° or more. There were valgus in two cases and varus deformity in two cases with angulation below 5°. In Group 2, there were 2 cases with varus angulation of 5° or more. There was varus in one case and valgus deformity in one case below 5°. There was no post-operative fibular angulation in Group 1 whereas there were two cases in Group 2. The presence of syndesmosis malalignment measured by post-operative computed tomography was found in five cases in Group 1 and 4 cases in Group 2 (p=0.874) (Table III).

The mean union time was 5.1 months (range: 4-10 months) in Group 1 and 5.1 months (range: 3-10 months) in Group 2 (p=0.857). There was no need for graft or flap due to skin necrosis in both groups (p>0.05). Reflex sympathetic dystrophy was seen in one case in Group 1 and two cases in Group 2. The two infected patients in Group 1 were hospitalised for deep infection and received intravenous antibiotic treatment. Group 2 had two superficial infections which responded to oral antibiotherapy. There was no difference in infection rates between two groups (p=0.894). There was no statically difference between the deep infections (p=0.175) and superficial infections (p=0.115) between the groups. One of the cases having a deep infection was 54-year-old hypertension patient without any relatives. The patient was staying in a nursing home and had a low quality of self-care. The other case was a 45-year old patient without any additional diseases. He had only the history of smoking.

## Discussion

IDTF fractures are high-energy injuries which may accompany severe soft tissue damage and increase complication rates^7,15^. The soft tissue layer on the distal tibia is thin and has limited vascularisation. This increases the tendency to such complications as wound problems, infections, non-union, and delayed union in distal tibial fractures^16,17^. As a result, the characteristics of soft tissue injury become an important condition affecting fracture outcomes and restricting the feasibility of osteosynthesis^18^. In fact, the most important factors in ensuring reduced rate of wound complications may be the recognition of soft tissue injuries and application of a surgical procedure considering timing and soft tissue healing. Most surgeons prefer a two-stage protocol designed to support healing of the traumatised soft tissue layer before definite fixation. The first stage of this surgical protocol focuses on the healing of the soft tissue component. The second stage is rather performed for definitive reduction and stabilisation of the joint surface and bone alignment^8^. It is believed that the two-stage surgical procedure is an effective method in order to decrease the complications in IDTF fractures involving soft tissue injury.

While osteosynthesis of the fibula is recommended with plate-screw in fibula-involved intra-articular distal tibia fractures, the preference of the stage where the fibula needs to be fixed in two-stage procedure is still controversial^2,9-12^. Advocates of the initial stage primarily believe that most of the tibial deformity could be reduced by restoration of correct fibular length, alignment and rotation and indirectly by ligamentotaxis. The also note that a stabilised fibula will support medial-based external fixator in reduction and stabilisation. Moreover, anatomical reduction of fibula could provide indirect reduction of anterior (Chaput) and posterior (Volkmann) tibial joint fragments associated with anterior and posterior distal tibiofibular syndesmotic ligaments, respectively^8,19^. In the present study, there was no difference between the two groups in terms of total surgical time, and the rates of post-operative deformities, intra-articular step-off, and syndesmosis malalignment. This might be an evidence of the fact that implementation of the fibula fixation at the first stage may not facilitate joint reduction, syndesmotic reduction or surgical reduction during definitive treatment.

On the other hand, advocates of the second-stage reduction state that such fixation could make anatomic tibia reduction impossible in case that fibula fracture is comminuted or in case of a bone defect^12^. They also highlight that application of the plate at the initial stage could compel posterior malleolar reduction and prevent lateral radiographic evaluation during the second stage of the surgery^20^. In the present study, no differences was found between the two groups in terms of post-operative fibular shortening, fibular rotation, and fibular angulation. There was no difference in post-operative tibial deformities as well.

Even, numerically; post-operative fibular shortening was found in one patient in Group 1 and three patients in Group 2. These results reveal that fixing the fibula in the first stage could be possible in contrary to what have been claimed; may occur with a result of not having a negative effect on tibial reduction.

Another claim of the advocates of the second-stage protocol was that inappropriate or unplanned skin incision at the first stage might negatively affect planning of the second skin incision or increase complication rates by posing a risk on the soft tissue due to presence of an insufficient skin bridge^11,12^. Some authors have noted that the distance between the two incisions should be at least 7cm in order to minimise the soft tissue complications in the surgical management of intra-articular distal tibia fractures^21,22^. Another study have presented that intra-articular distal tibia fractures of 7cm under the skin bridge in contrary to the claims to be associated with low soft tissue complications^22^. In our study, there was no difference between the two groups in terms of soft tissue necrosis, infection and skin flap/graft requirement. No skin necrosis was observed in the groups. No data were obtained about skin bridge thickness between two incisions in our study. Contrary to the general assumption, it could be noted that the safe length of skin bridge could be below 7cm in intra-articular distal tibia fractures. The application of the fibular plate through a posterolateral skin incision may be sufficient to provide a safe skin bridge.

Orthopaedic surgical approaches may be affected by surgeons’ training level or habits in their clinics^23,24^. Chan *et al* reported that there was no significant difference between preference of the initial or second stage for fibula plate in terms of tibia-fibula union time, sagittal-coronal plan angulation, and surgical duration^5^. It may not be significant to identify which stage the fibula is fixed in the two-stage protocol for IDTF fractures. The reason for the preference of either the first or the second stage may be attributed to surgical opinions or habits. The fact that there was no significant difference between the two groups in our study may support this idea.

Deep infections in IDTF fractures are one of the complications that have been repeatedly reported by many researchers^1^. Joveniaux *et al* reported that there was no correlation between infection and fracture severity, type of osteosynthesis, surgical approach, or initial skin damage^2^. Another study suggested that fixing the fibula early or late had no effect on deep and superficial infection rates^5^. In the present study, there was no statistical significance between deep and superficial infection rates between the two groups. However, both cases with deep infection were cases where fibula plate was applied in the first stage. It is believed that this might be related to comorbid factors that patients have. Perhaps, the fact that several cases of infection were collected in a group may have caused this situation due to the number of cases included in the study.

The present study has several limitations. The first limitation is its retrospective nature. Other shortcomings include no information about the lengths of the skin bridges and no comparison of rates post-operative malalignment to the preoperative syndesmotic malalignment due to the lack of the latter.

## Conclusion

In two-stage surgical procedures in IDTF fractures, postoperative complications, radiographic and functional results might be comparable between cases where fibular fixation was performed during temporary surgery and the cases where it was performed during permanent surgery. The application of the fibula plate in the first stage does not increase soft tissue complications. However, it may not provide any superiority in terms of operation times, intra-articular step-off, tibial deformities, AOFAS scores and syndesmosis malreduction rates. It could be noted that the timing of fibular fixation in two-stage surgery in IDTF fractures should be left to the surgeon's own discretion with a good surgical panel.
